# Acute Restraint Stress Impairs Aversive Memory Retention but Not Memory Formation

**DOI:** 10.3390/biology14091204

**Published:** 2025-09-06

**Authors:** Aline Lima Dierschnabel, Diana Aline Nôga, Luiz Eduardo Mateus Brandão, Catherine Caldas de Mesquita, Diego de Aquino Câmara, Ywlliane da Silva Rodrigues Meurer, Felipe Porto Fiuza, Rovena Clara Galvão Januário Engelberth, Regina Helena da Silva, Jeferson Souza Cavalcante, Ramón Hypolito Lima

**Affiliations:** 1 Laboratory of Neurochemical Studies, Physiology and Behaviour Department, Post-Graduation Program in Psychobiology, Federal University of Rio Grande do Norte (UFRN), Natal 59078-970, Brazil; 2 Department of Pharmaceutical Biosciences, Uppsala University, SE-751 05 Uppsala, Sweden; 3 Department of Medical Sciences, Uppsala University, SE-751 05 Uppsala, Sweden; 4 Santos Dumont Institute, Edmond and Lily Safra International Institute of Neuroscience, Macaíba 59280-000, Brazil; 5 Behavioural Neuroscience Laboratory, Department of Pharmacology, Universidade Federal de São Paulo (EPM/UNIFESP), São Paulo 04021-001, Brazil

**Keywords:** aversive memory retention, stress, hippocampus, Zif268, C-fos, plus-maze discriminative avoidance task

## Abstract

Stress can alter neurochemical signalling, affecting memory processing, but its underlying neurobiological mechanism remains unclear. Here, we investigate the effect of acute restraint stress (ARS) on long-term retention of aversive memory in rats. We exposed the animals to either handling or an ARS protocol and tested the rats in the plus-maze discriminative avoidance task (PMDAT). Also, we performed immunohistochemistry assays to unveil the effect of stress on neuronal activity. We found that ARS immediately after training does not impair memory formation but hinders retention. Training triggers a peak of C-fos 1 h later and a delayed 18 h increase in Zif268 in dorsal CA1. The same does not occur when ARS is experienced immediately after training. We demonstrated that the crucial role of Zif268 and C-fos signalling in maintaining PMDAT LTM. ARS is more relevant for memory retention than for the formation of discriminative aversive memory.

## Simple Summary

Stress is a common experience that can influence how memories are stored and remembered. In this study, we explored how acute restraint stress, applied after learning, affects memory. Rats were trained in a task where they had to learn to avoid an unpleasant situation. After training we tested the animals to see how long they remembered what they had learned. We found that stress did not stop the rats from forming the memory, but it did make the memory fade more quickly over time. This effect was linked to a reduced activity of brain signals that normally help memories last. These findings show how stress can weaken memory retention and may provide insights into stress-related memory retention.

## 1. Introduction

Stressful events are an inherent part of life, with the potential to either promote resilience and personal growth or contribute to stress-related disorders. Although individuals may experience similar events, their responses vary, highlighting the diverse ways in which organisms adapt to adversity [1,2].

The behavioural outcomes of stress exposure depend on how experience-dependent life events are acquired and stored. Stress can either impair or enhance memory function depending on various factors, including the nature of the stimuli, intensity, duration, controllability, timing within the memory processing phase, and the type of memory involved [3].

Research findings on the effects of acute restraint stress (ARS) on memory are conflicting. Some studies report that ARS before learning disrupts mnemonic trace stability and impairs memory acquisition [4,5,6,7], while others show enhanced learning, particularly for emotional content [8,9]. Similar discrepancies exist for memory consolidation, with stress either strengthening or impairing it, depending on factors such as intensity and duration.

These opposing outcomes result from changes in neuronal activity within limbic regions, such as the hippocampus [10]. Stress regulates cognitive functions and increases long-term memory (LTM) protein synthesis in the hippocampus [11,12,13].

Dynamic neuronal gene expression, influenced by fluctuations in neuronal activity, supports neuronal plasticity. Hippocampal immediate-early genes (IEGs), such as C-fos and Zif268, activate and translate in neurons involved in learning processing [14]. Evidence suggests that upregulation of C-fos and Zif268 expression in the dorsal hippocampus underlies LTM formation, consolidation, and storage [15,16,17,18]. The hippocampus is sensitive to stress, and dysregulation of C-fos expression in this region has been linked to stress-related disorders, including depression and anxiety [19,20].

We investigate the impact of post-training ARS on the maintenance of aversive memory and the expression of C-fos and Zif268 in rats subjected to the plus-maze discriminative avoidance task (PMDAT). This task assesses memory, anxiety, and locomotor activity [21].

Understanding how acute stress impacts memory retention is crucial for elucidating the neural mechanisms underlying adaptive and maladaptive responses to stress. While previous studies have explored the effects of stress on learning, the temporal dynamics of post-training stress and its specific influence on memory retention remain poorly understood. Our study adds information to the literature by examining how a single episode of acute restraint stress immediately after training affects PMDAT memory retention over time and neuronal activation, providing new insights into the interplay between stress and memory processes.

## 2. Materials and Methods

### 2.1. Subjects and Experimental Procedures

Subjects were 128 Three-month-old male Wistar rats weighing 280–300 g. Animals were housed in home cages (4–5 animals per cage) under controlled conditions of temperature (22–25 °C), humidity, with food/water at will and a light-dark cycle of 12 h/12 h (lights on 6:30 a.m.). All animals were handled according to Brazilian law for the use of animals in scientific research (Law Number 11.794), and all procedures were approved by the local ethics committee (CEUA/UFRN—Nº 039/2014). Animals were randomly assigned to each group.

### 2.2. Behavioural Apparatus—Plus-Maze Discriminative Avoidance Task (PMDAT)

This apparatus consists of a modification of the elevated plus-maze (50 × 15 ×40 cm): two open arms (OA) opposite to two enclosed arms—a non-aversive enclosed arm (NAV) and an aversive enclosed arm (AV). In the training session, we placed animals in the centre of the maze, allowing them to explore the apparatus for 10 min. Every time animals entered the AV, an aversive stimulus (100 W of light plus 80 dB of noise) was fired until the animal left the arm.

To evaluate memory retention in the PMDAT, animals were trained and subsequently divided into a control group (n = 7–9 per group) and an ARS group (n = 8–9 per group). Both groups were tested at 1, 3, 5, and 7 days after training to assess the influence of stress on long-term memory retention.

### 2.3. Acute Restraint Stress (ARS) Protocol

We exposed the animals to either handling or a 1 h ARS protocol in a separate laboratory room. The apparatus consisted of an acrylic tube (6.5 × 19.8 cm) with ventilation holes to ensure proper breathing during exposure. Behavioural tests were conducted 24 h after handling or ARS exposure. However, for immunohistochemistry experiments, animals were exposed to different conditions and euthanised at two time points, as described below. Animals were randomly assigned to each group.

### 2.4. Immunohistochemistry

To investigate the expression patterns of Zif268 and c-Fos following PMDAT, animals were euthanised and perfused at specific time points (n = 3–4 rats per group). The experimental groups included: (1) naïve animals, (2) naïve stress (rats exposed to the AV with light-noise pairings—five trials of a 3 s stimulus with a 5 s interval), (3) animals euthanized 1 h after handling or (4) after ARS exposure, and (5) animals euthanized 18 h after handling or (6) after ARS exposure.

After appropriate manipulation of each experimental group, rats were anaesthetised with thiopental (100 mg/kg) and transcardially perfused with phosphate-buffered saline (PBS), pH 7.4, followed by 4% paraformaldehyde in 0.1 M phosphate buffer, pH 7.4. Each brain was sectioned into 50 µm slices with a cryostat microtome (Leica, Wetzlar, Germany) at −22 °C and placed sequentially in six series with an anti-freezing solution. The distance between one section and the next in the same series was approximately 300 µm.

To detect Zif268 and C-fos proteins, we performed an ABC-free-floating immunohistochemistry protocol. Sections were incubated for 16–18 h with anti-C-fos (Santa Cruz Biotechnology [sc-52], 1:1000 Dallas, TX, USA) or anti-Zif268 (Santa Cruz Biotechnology [sc-189], 1:1000 Dallas, TX, USA) primary antibodies.

The solution contained 1% albumin diluted in 0.4% Triton X-100 and 0.1 M phosphate buffer, pH 7.4, and then incubated with a biotinylated secondary anti-rabbit antibody (Jackson ImmunoResearch Laboratories Inc.,West Grove, PI, USA, 1:1000) for 2 h. Slices were washed and incubated with avidin-biotin-peroxidase solution (ABC Elite kit, Vector Labs, Burlingame, CA, USA) for 2 h. The reaction was developed by adding diaminobenzidine tetrahydrochloride (Sigma, St. Louis, MO, USA) and 0.3% H_2_O_2_ in 0.1 M phosphate buffer, pH 7.4. Sections were washed, dried, dehydrated in a graded alcohol series, cleared in xylene, and coverslipped with Entellan (Merck Rahway, NJ, USA).

All immunostainings were performed concomitantly, minimising possible differences in background among animals. Sections were examined by using an optical microscope (Nikon Eclipse Ni-U, Nikon, Nishioi, Shinagawa-ku, Tokyo, Japan) with a digital camera (Nikon, DS-Ri2, Nishioi, Shinagawa-ku, Tokyo, Japan) to record images.

To estimate the density of Zif268- and C-fos-positive cells in the pyramidal layer of dorsal hippocampus, we systematically selected four sections from each animal. Using the rat brain atlas [22], we identified brain sections located between −3.24 mm and −4.2 mm from the bregma. We analysed immunoreactive cells and measured hippocampal subregion areas with ImageJ software (version 1.48, NIH, Madison, WI, USA).

### 2.5. Statistical Analysis

Memory persistence in the PMDAT was evaluated by comparing total time (s) spent in the AV and NAVs, and the percentage of total time spent in the AV [%TAV = time in AV/(time in NAV + AV) × 100]. Anxiety-like behaviour was evaluated by the percentage of time spent in open arms [% TOA = time in OA/(time in OA + NAV + AV) × 100] in training and test sessions. Locomotor activity was evaluated by total distance travelled (m) in training and test sessions.

We analysed the data using GraphPad Prism 9 and applied one-sample *t*-tests, two-way ANOVA followed by Tukey’s post hoc test, or one-way ANOVA followed by Tukey’s or Bonferroni’s post hoc test, depending on the experimental requirements. We set statistical significance at *p* < 0.05.

## 3. Results

**Figure 1:**
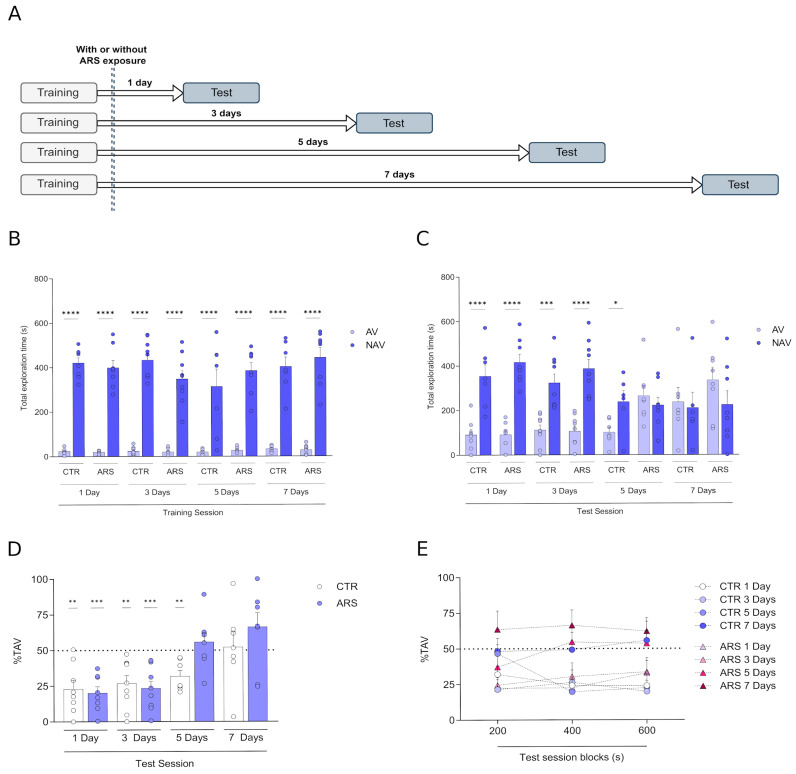
Memory retention in the plus-maze discriminative avoidance task (PMDAT). (**A**) Schematic diagram of experimental setup. Comparisons between total time spent in AV and NAVs in (**B**) training and (**C**) test sessions. Percentage of total time spent in the aversive enclosed arm (%TAV) in the whole session (**D**) and in blocks of 200 s (**E**). Data expressed as mean ± SEM. (**B**,**C**) After Tukey’s post hoc test: **** *p* < 0.0001; *** *p* < 0.001; * *p* < 0.05, comparing AV vs. NAV in all groups. (**D**) ** *p* < 0.01 and *** *p* < 0.001 comparing groups with a 50% chance of exploration of each arm (one-sample *t*-test). (**E**) Groups were compared among blocks (RM two-way ANOVA followed by Tukey’s post hoc test).

Our results indicate that animals submitted to the PMDAT responded to the aversive stimulus throughout the training session and preferred the NAV-enclosed arm (Figure 1B).

In the test sessions, the control group showed a statistically significant preference for exploring the non-aversive enclosed arm for up to 5 days after training, whereas the ARS group showed this preference only up to 3 days after training [Figure 1C; interaction—F_(7,109)_ = 8.017; groups—F_(7,109)_ = 1.078; exploration F_(1,109)_ = 36.97; followed by Tukey’s post hoc correction, **** *p* < 0.0001; *** *p* < 0.001; * *p* < 0.05].

When the percentage of time spent in the aversive enclosed arm was evaluated as a discriminatory proxy we found that control animals were able to discriminate enclosed arms for up to 5 days [Figure 1D; 1 day (t_(7)_ = 4.393; *p* < 0.001), 3 days (t_(8)_ = 4.098; *p* < 0.001) or 5 days (t_(6)_ = 4.544; *p* < 0.001)]; however, we observed that ARS exposed rats showed an impairment of PMDAT memory retention compared to control group [Figure 1D; 1 day: t_(7)_ = 6.675; *p* < 0.001; 3 days: t_(8)_ = 5.429; *p* < 0.001; 5 days: t_(7)_ = 0.91; *p* = 0.393; 7 days: t_(6)_ = 1.675; *p* = 0.1379].

When we analysed the control group across time in the test session (blocks of 200 s), the 7 days group showed higher AV exploration in the last block when compared to all groups [Figure 1E; interaction—F_(6,54)_ = 3.017; blocks—F_(2,54)_ = 2.879; retention time F_(3,27)_ = 3.175; followed by Tukey’s post hoc correction, * *p* < 0.05].

Moreover, we found that ARS animals showed a similar output when we analysed the test session in blocks of 200 s; however, the 7 Days group is different from the 1 and 3 Days groups in the first block and from the 1 Day group in the second block [Figure 1E; interaction—F_(6,62)_ = 1.153; blocks—F_(2,62)_ = 3.721; retention time—F_(3,31)_ = 3.412; followed by Tukey’s post hoc correction, * *p* < 0.05].

**Figure 2:**
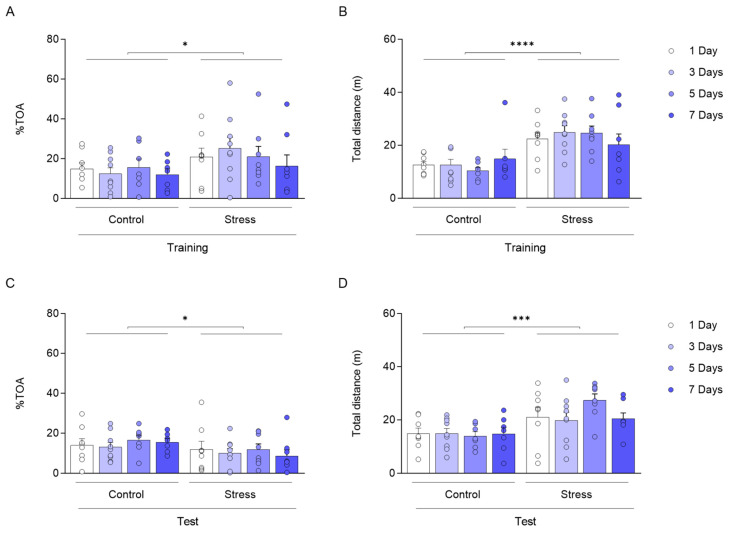
Anxiety-related behaviour and total exploration in PMDAT are not altered by training or ARS exposure. Percentage of time spent in open arms (%TOA) in the training session (**A**) and test session (**C**). Total distance travelled (m) in training session (**B**) and in the test session (**D**) for control and ARS animals, subsequently. Data expressed as mean ± SEM). (**A**,**C**) * *p* < 0.05 comparing groups; (**B**) **** *p* < 0.0001; (**D**) *** *p* < 0.001. All comparisons were made using Two-way ANOVA with test days after training as a second factor.

We found changes in anxiety-like behaviour in training [Figure 2A; F_(1,56)_ = 5.128; * *p =* 0.0274) and test (Figure 2C; F_(1,56)_ = 4.317; * *p* = 0.0423)], and exploratory behaviour comparing ARS groups to control groups during training [(Figure 2B; F_(1,56)_ = 30.83; **** *p* < 0.0001) and test (Figure 2D; F_(1,56)_ = 17.44; *** *p* < 0.001)].

**Figure 3:**
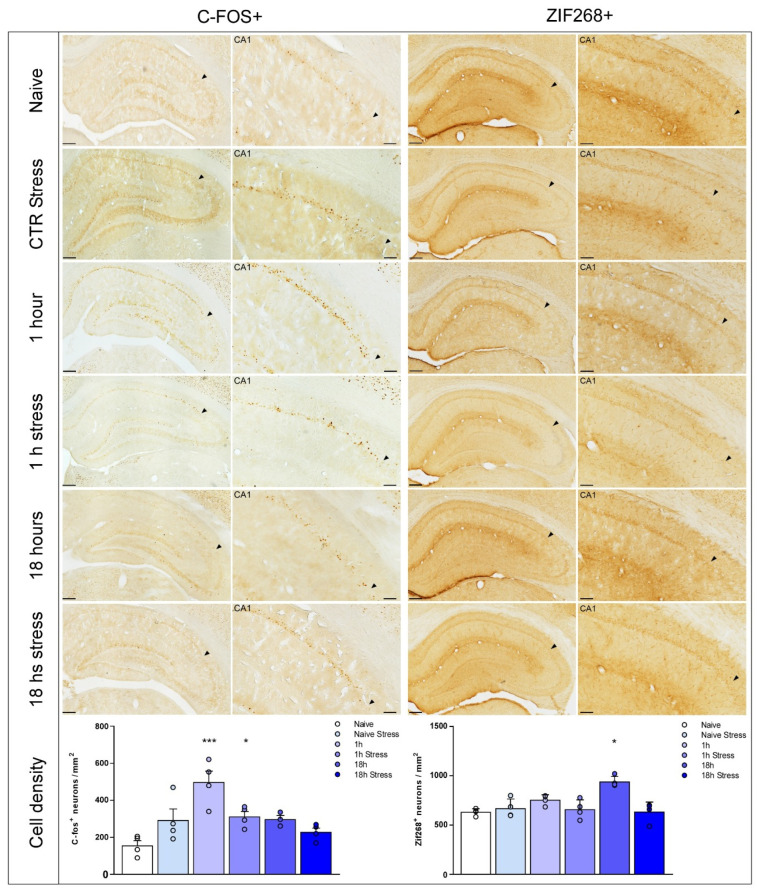
ARS immediately after training reverses C-Fos and Zif268 expression in the CA1 region of the dorsal hippocampus. C-fos (**left**) and Zif268 (**right**) immunoreactivities at the CA1 region of the dorsal hippocampus are indicated in each row for the distinct groups. The CA1 region is highlighted in higher magnification in the images on the right. Black arrows point to the limit between CA1 and CA2 hippocampal subregions. C-fos ((**Bottom left**) graph) and Zif268 (**Bottom right**) cell densities in the CA1 region of the dorsal hippocampus are shown at different time points after training in rats exposed to ARS and its respective control groups. Data expressed as mean ± SEM. Asterisks indicate the comparison with the naive group (one-way ANOVA followed by Bonferroni’s post hoc test). Scale bar = 250 μm (dorsal hippocampus) and 100 μm (CA1 regions highlighted) in 100× magnification.

Our results show that ARS exposure prevented the increase in the cell density of C-Fos at 1 h [Figure 3; F_(5,17)_ = 7.713; *p* = 0.0006] and Zif268 at 18 h [Figure 3; F_(5,18)_ = 9.580; *p* = 0.0001] after training in the CA1 subregion of the dorsal hippocampus. After multiple comparisons by Bonferroni’s post hoc test, * *p* < 0.05 and *** *p* < 0.001 when compared to the naive group.

## 4. Discussion

The importance of our work relies upon understanding how acute stress modulates the hippocampal activity required to store information in long-term memories. We show that animals form and retrieve a persistent and reliable memory up to five days after training. This outcome allowed us to investigate the role of acute stress on the animal’s capacity to hold information about aversive memories. Other behavioural tasks can form stronger memories through electroshocks on rats’ paws as aversive stimuli [23,24], but would jeopardise our ability to distinguish learning from stress induction.

The PMDAT requires both amygdala and dorsal hippocampus activity for memory formation and retrieval [25,26]. Nevertheless, the role of hippocampal subregions in the retention of PMDAT memory still needs to be understood.

The hippocampal subregions are often associated with different roles in memory formation and other facets. The CA3 subregion is thought to be involved in spatial memory and pattern separation, while DG is associated with context discrimination [27,28]. On the other hand, the CA1 subregion plays a crucial role in memory formation and persistence of LTMs [24,29]. It has also been suggested to be the primary output from the hippocampus to cortical areas [30,31].

Immediate-early genes (IEGs) regulate a wide range of genes and proteins and are often used to assess hippocampal activity levels [32,33,34]. C-fos and Zif268 have an essential role in the regulation of synaptic formation, transmission, and plasticity as well as memory processing [35,36,37,38,39]. IEGs upregulation in dorsal hippocampus pyramidal neurons underlies aversive memories’ long-lasting storage through MAPK/ERK pathway activation [17,40,41,42]. Multiple reports showed that C-fos and Zif268 inhibition impairs the retention of aversive memories by reducing BDNF expression [40,43], emphasising their relevance to this phenomenon.

C-fos and Zif268 are suggested to display a biphasic expression; an early peak, responsible for memory formation, and a late peak responsible for memory persistence [15,40,43]. We found that Zif268 and C-fos cell density increased later after training in CA1. Contrariwise, only C-fos levels increased in other hippocampal regions, suggesting C-fos is sensitive to environmental stimuli such as novelty. Our results strengthen the idea that memory maintenance involves a late rather than early peak of Zif268 synthesis [16].

Acute stress promotes changes in hippocampal activity, impairing STM and LTM retrieval but enhancing LTM consolidation in humans and rodents. However, the effect varies depending on the nature of the events that trigger memory formation [44,45,46,47,48,49,50,51,52,53,54,55,56]. We found that ARS immediately after training did not alter memory formation, but reduced retention of PMDAT. Taken together, ARS differentially modulates LTM processing, hindering the persistence of LTM without affecting memory consolidation. However, neurochemical signalling and neural modifications underlying these behavioural changes are yet to be solved.

We repeated the neurochemical experiment to assess the effect of stress in either early (C-Fos) or late (Zif268) expression. We found that Zif268 cell density does not increase in non-stressed animals euthanised 18 h after training. Moreover, stress did not cause any changes in animals euthanised 1 h after training, suggesting that changes in Zif268 levels are related to retention of PMDAT memory. Conversely, we found the opposite results for C-Fos immunolabeling. Therewith, we did not find a stress effect perse, as naive animals submitted to stress induction did not show any disturbance in C-Fos and Zif268 levels.

Studies showed that ARS inhibits synaptic plasticity in the hippocampus-prefrontal cortex circuit in rodents [57]. Additionally, stress reduces the synthesis of other proteins for memory persistence, such as CREB, BDNF, C-Fos, and Zif268 [11,58,59,60]. Collectively, one could argue that stress triggers an increase in corticosterone release in the hippocampus, modulating protein expression through its interaction with glucocorticoids and mineralocorticoids receptors, which could undermine LTM retention and long-term potentiation (LTP) maintenance [17,61].

Based on this rationale, increasing IEG synthesis in the hippocampus to promote the retention of PMDAT LTM [62,63] corroborates our results. Also, ARS immediately, but not late after training, decreases Zif268 expression in the same brain region [64].

## 5. Conclusions

In conclusion, we show the role of Zif268 and C-fos signalling in the formation and retention of PMDAT LTM. Both IEGs increase their expression in the hippocampus late after training. However, Zif268 overexpression has a high specificity in the dorsal hippocampus CA1 subregion, while C-fos increase its activity in CA1 and CA3. We show that acute restraint stress immediately after training harms the maintenance of PMDAT memory and down-regulates Zif268 expression in CA1. Our results will help unveil the mechanisms underlying LTM maintenance under acute stress modulation. Persistence of an aversive memory and the stress situation surrounding learning play a key role in the development of psychiatric disorders, such as post-traumatic stress disorders. In other words, our results will benefit a broad range of studies, from basic research to clinical trials.

Our study provides important insights into how acute post-training stress affects memory retention and neuronal activation. Future studies should explore different stress timings or durations, incorporate endocrine markers, and examine additional molecular pathways to further clarify the mechanisms by which acute stress influences memory formation, consolidation, retention, reconsolidation, and extinction. These efforts will help to establish a more comprehensive understanding of the interplay between acute stress, neuronal activity, and memory processing, potentially guiding the development of interventions to mitigate stress-related cognitive impairments.

## Data Availability

The datasets generated and analysed during the current study are available from the corresponding author on reasonable request.
